# Morning glory syndrome associated with multiple sclerosis

**Published:** 2014-07-04

**Authors:** Anahid Safari, Esmail Jafari, Afshin Borhani-Haghighi

**Affiliations:** 1 Clinical Neurology Research Center, School of Medicine, Shiraz University of Medical Sciences, Shiraz, Iran; 2 Department of Ophthalmology, Division of Corneal Disease, School of Medicine, Iran University of Medical Sciences, Tehran, Iran; 3 Department of Neurology, School of Medicine AND Clinical Neurology Research Center, Shiraz University of Medical Sciences, Shiraz, Iran

**Keywords:** Morning Glory Syndrome, Morning Glory Disc Anomaly, Multiple Sclerosis, Demyelinative Disorders, Optic Neuritis

## Abstract

Morning glory syndrome (MGS) is a rare congenital optic disc anomaly characterized by a funnel-shaped, excavated optic disc surrounded by chorioretinal pigmentary disturbance. The main ophthalomoscopic feature of the MGS is enlarged optic disc with a funnel shaped scleral defect; elevated peripapillary chorioretinal pigmentation; and pale fluffy tissue of glial hyperplasia overlying the optic disc. Although most of the reported cases were isolated ocular abnormality, but it may occurs in association with other ophthalmic abnormalities such as cyst of the optic nerve atrophy, congenital cataract, microophthalmos, and aniridia. Craniofacial deformities such as cleft lip and palate, hypertelorism, dysplatic ears; renal abnormalities; and cardiac defects have also been reported with MGS. Herein, we present a case of MGS associated with multiple sclerosis - a rather unusual concurrence.

## Introduction

Morning glory syndrome (MGS) is a rare congenital optic disc anomaly characterized by a funnel-shaped, excavated optic disc surrounded by chorioretinal pigmentary disturbance.^1^ The name MGS derives from the resemblance of the optic disc to the trumpet shaped morning glory flower.^2^ Although most of the reported cases were isolated ocular abnormality, but it may occurs in association with other systemic manifestation.^2^^-^^14^ Herein, we present a patient with MGS who had attacks of paresthesia, urinary frequency, and optic neuritis. Magnetic resonance imaging (MRI) fulfilled McDonald criteria of multiple sclerosis (MS) consequently, she was considered to have both MS and MGS - a rather unusual concurrence.

## Case Report

A 27-year-old Iranian woman presented with blurred vision of the left eye, paresthesia, and severe sense of fatigue 10 days prior to admission. She was admitted to the Neurology ward of Nemazee Hospital affiliated to Shiraz University of Medical Sciences, Shiraz, South of Iran.

The patient was relatively well until 1 year post-traumatic amnesia (PTA), when she developed tingling and numbness of the left half of the face, and urinary frequency and urgency for about 1 week. Thereafter, she had other bouts of vertigo, ataxia, and voice change. These episodes durated about a few days and improved spontaneously. She also complained of numbness, muscle soreness, and tightness, early fatigue since 1 year PTA.

She suffered from progressive visual loss of the right eye since 9 years old. Its cause had not been known 2 years PTA that MGS was diagnosed for her.

**Figure 1:**
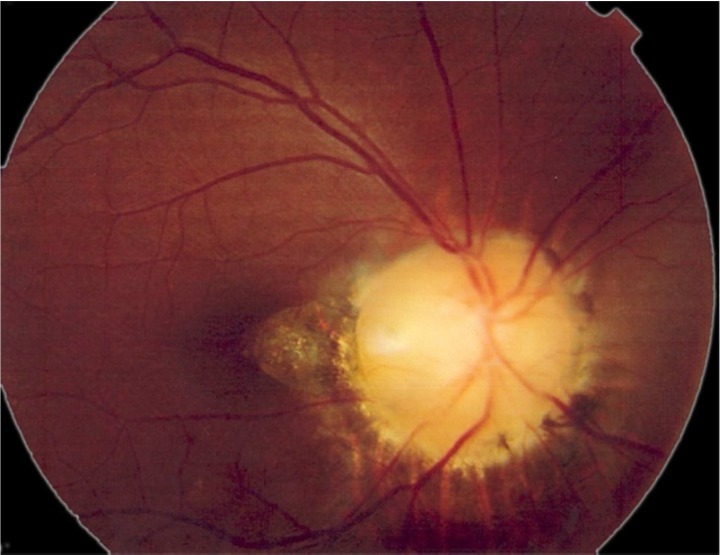
Right optic fundus of the patient showing an enlarged whitish optic disc with funnel shaped scleral defect and a pigmented peripapillary annulus (Morning glory phenomenon)

General physical examination was unremarkable. Ophthalmologic examination revealed best corrected visual activity (VA) VA20/200 in the right eye (RE) and 20/20 in the left eye (LE), relative afferent pupillary defect: +2 RE, manifest and cycloplegic refraction were Plano in both eyes, and slit-lamp examination was normal and Goldman applanation tonometry: 13 mmHg in RE and 12 mmHg in LE. Right optic fundus revealed an enlarged whitish optic disc with funnel shaped scleral defect and a pigmented peripapillary annulus (Figure 1).

In neurologic examination, she was conscious and oriented. The patient had only very mild clumsiness in fine movements of the right hand, but muscle power of all detected muscles were 5/5 in Medical Research Council scale. Plantar reflexes were downward on both sides. Deep tendon reflexes were increased to 3/4 on both sides. In cerebellar examinations, she had impairment of tandem gait, but normal finger to finger and heel to shin tests. In sensory examination, she had mild impairment of the sensation of vibration in lower extremities. She had no neck rigidity.

Routine biochemistries, complete blood count, urinalysis, stool examinations for occult blood/parasite, liver function test, thyroid function test, lipid profiles, and creatine kinase level were all normal. Tests for anti-human immune deficiency virus antibodies, hepatitis B surface antigen, and hepatitis C virus antigen were also negative. Rheumatoid factor, anti-nuclear antibody, anti-double strand DNA antibody, anticardiolipin antibodies, anti-neutrophil cytoplasmic antibodies, and syphilitic serology tests were also unremarkable.

**Figure 2:**
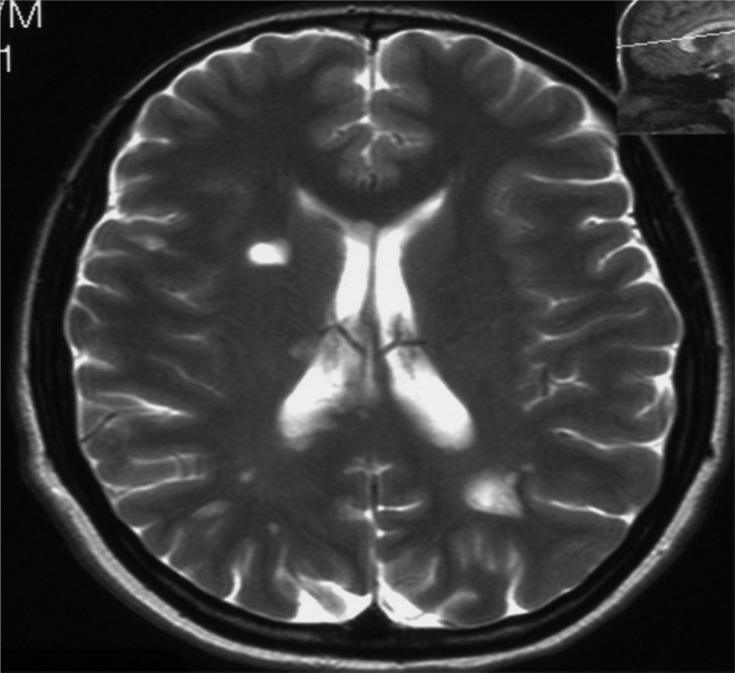
Brain magnetic resonance imaging, T_2_-weighted image showing hyper-signal demyelinating plaques in the periventricular and deep white matter areas

**Figure 3:**
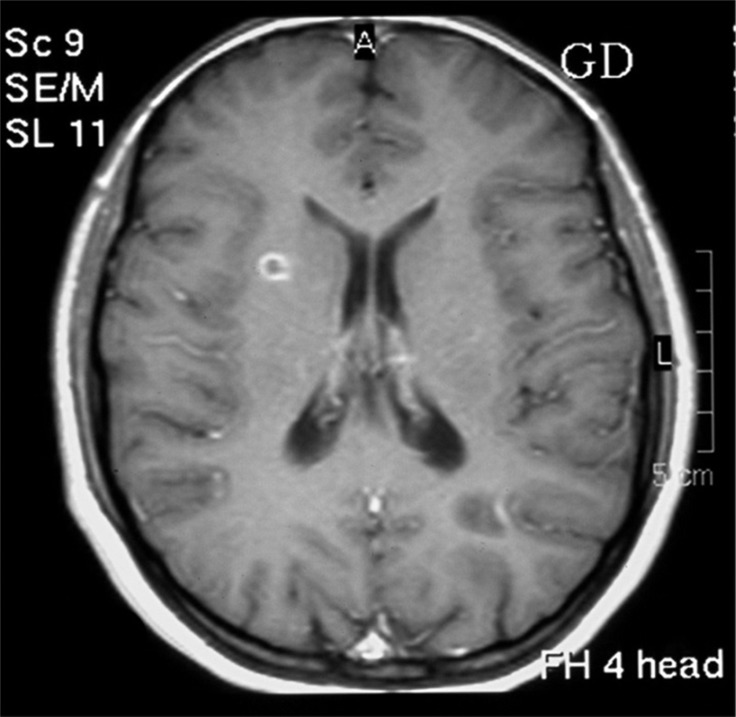
Brain magnetic resonance imaging, T_1_-weighted image showing ring-like enhancement of the active plaque

Brain MRI showed about 5-6 hyper-attenuated ovoid and round lesions in the periventricular and subcortical areas in T_2_-weighted and fluid attenuated inversion recovery images (Figure 2). These lesions were isointense in T_1_-weighted images. The largest lesion revealed a ring-like pattern of enhancement after administration of gadolinium (Figure 3). Meanwhile, MRI of the cervical spine showed one hypersignal lesion in the posterior column at the level of C4-C5.

Intravenous methylprednisolone, 1000 mg/24 h for 5 days followed by 30 mg prednisolone per os (by mouth) twice daily was prescribed for the patient. Two to three days after administration of methylprednisolone, vision of the left eye improved significantly. Amantadine 100 mg twice daily, gabapentin 300 mg twice daily, citalopram 20 mg once a day, and terazosin 1 mg at every bed time were prescribed for treatments of different symptoms. Another brain MRI was conducted 6 months later, which revealed two new T2 lesion. Accordingly, she fulfilled both dissemination in time and dissemination in space McDonald criteria of MS. The patient was considered as a case of MS and weekly ß-interferon 1-a (Avonex) was prescribed for her. Informed consent was obtained from her for this report.

## Discussion

We reported a patient with MGS who developed several episodes of multifocal neurological disorders since 1 year PTA. She can be considered a case of clinically definite relapsing-remitting MS (Fulfilling both Poser’s criteria^15^ and McDonald criteria^16^).

MGS is a rare congenital anomaly of the optic papilla. Although, MGS is often an isolated ocular abnormality; however, it may be associated with systemic abnormalities.

The main ophthalmoscopic feature of the MGS is enlarged optic disc with a funnel shaped scleral defect; elevated peripapillary chorioretinal pigmentation; and pale fluffy tissue of glial hyperplasia overlying the optic disc.^1^ No definite pattern of inheritance has been found for MGS.^3^ In children, the male to female ratio is about 1:2.^4^ The majority of cases are unilateral, but some bilateral cases are reported.^5^ The exact pathogenesis of the MGS is unknown yet. A combined defect of ectodermal and mesodermal dysgenesis has been proposed as the probable mechanism.^6^

Less than 100 cases of MGS have been reported in the medical literature. It may be an isolated phenomenon or associated with other ophthalmic abnormalities such as cyst of the optic nerve sheath,^7^ optic nerve atrophy,^3^ congenital cataract,^8^ microophthalmos,^9^ and aniridia.^9^ Craniofacial deformities such as cleft lip and palate,^3^^,^^10^ hypertelorism,^3^ dysplatic ears;^7^ renal abnormalities such as renal hypolpasia and chronic glumerulonephritis,^10^ hydronephrosis;^11^ and cardiac defects^7^ have also been reported with MGS.

The reported neurologic associations include agenesis of the corpus callosum,^3^^,^^11^^,^^14^ porencephaly, ^11^ brain atrophy,^11^ hypopituitarism,^2^^,^^12^ basal myelomeningocele, encephalocele,^3^^,^^10^^,^^12^ and facial palsy.^7^ Association with CHARGE syndrome (Coloboma, Heart disease, Atresia choanae, Retarded growth, Genital anomalies, and Ear anomalies)^4^ and Poland syndrome (Absence of pectoralis muscle, arm hypoplasia, and Synbrachidactily)^13^ have also been published.

To the best of our knowledge, the association of MGS with MS has not been previously described. It is not clear which pathogenic mechanism play a role in coexistence of both MGS and MS. The presented case reinforces the need for exact evaluation of patient with MGS for early diagnosis and treatment of associated systemic anomalies. In addition, ophthalmologists should consider MS as one of possible neurologic associated disorder with MGS. 

## Notes

**How to cite this article: **Safari A, Jafari E, Borhani-Haghighi A. Morning glory syndrome associated with multiple sclerosis. Iran J Neurol 2014; 13(3): 177-80.
